# NOVEL intronic *CAPN3* Roma mutation alters splicing causing RNA mediated decay

**DOI:** 10.1002/acn3.50910

**Published:** 2019-10-14

**Authors:** Fabiola Mavillard, Marcos Madruga‐Garrido, Eloy Rivas, Emilia Servián‐Morilla, Rainiero Ávila‐Polo, Irene Marcos, Francisco J. Morón, Carmen Paradas, Macarena Cabrera‐Serrano

**Affiliations:** ^1^ Instituto de Biomedicina de Sevilla (IBiS) Hospital Universitario Virgen del Rocío/CSIC Universidad de Sevilla Sevilla Spain; ^2^ Centro Investigación Biomédica en Red Enfermedades Neurodegenerativas (CIBERNED) Instituto de salud Carlos III Sevilla Spain; ^3^ Neuromuscular Disorder Unit Pediatric Neurology Department Hospital U. Virgen del Rocío Sevilla Spain; ^4^ Department of Pathology Hospital U. Virgen del Rocío Sevilla Spain; ^5^ Department of Maternal‐Fetal Medicine, Genetics and Reproduction Hospital U. Virgen del Rocío Sevilla Spain; ^6^ Centro Investigación Biomédica en Red Enfermedades Raras (CIBERER) Seville Spain; ^7^ Department of Neurology Hospital Virgen del Rocío Sevilla Spain

## Abstract

*CAPN3* mutations cause a limb girdle muscular dystrophy. Functional characterization of novel mutations facilitates diagnosis of future cases. We have identified a novel (c.1992 + 2T>G) *CAPN3* mutation that disrupts the donor splice site of intron 17 splicing out exon 17, with mRNA levels severely reduced or undetectable. The mutation induces a strong change in the 3D structure of the mRNA which supports no‐go mRNA decay as the probable mechanism for RNA degradation. The mutation was identified in two unrelated Roma individuals showing a common ancestral origin and founder effect. This is the first Roma *CAPN3* mutation to be reported.

## Introduction

The Roma population is the most numerous ethnic minority in Europe. A small number of ancestors, subsequent endogamy, and population fissions causing secondary founder effects explain the increased frequency of Mendelian disorders in this population.^1^, ^2^, ^3^, ^4^, ^5^ Founder mutations are often found to cause 100% of the cases of a specific disease in this population group,^6^, ^7^ with important implications for diagnosis. Several neuromuscular disorders are particularly frequent among the Roma.^7^, ^8^, ^9^ The identification of founder mutations would allow a rapid diagnosis and prevention of future cases in this population group through genetic counselling.

*CAPN3* encodes a calcium modulated nonlysosomal protease predominantly expressed in skeletal muscle.^10^ Limb girdle muscle dystrophy 2A (LGMD2A), due to *CAPN3* mutations is one of the most frequent muscular dystrophies, however, its presence in the Roma population has not been reported before. Different mutation types have been reported in *CAPN3*, including splice site mutations.^12^ Here, we describe a novel intronic mutation that causes exclusion of exon 17 and RNA mediated decay.

## Patients and Methods

### Subjects of study

Findings from two nonrelated Iberian Roma subjects from Andalusia (Spain) are reported. The study was approved by the local Ethics Committee at Hospital Universitario Virgen del Rocío. Informed consent was obtained from the legal representative.

### Muscle histology

Muscle samples were obtained by open biopsy from both patients and processed following standard procedures. Usual stains for muscle and routine immunohistochemical stainnings (IHC) including utrofin (Novocastra Ref. DRP3/20C5), and calpain‐3 (Leica NCL‐CALP‐2C4 and Leica NCL‐CALP‐12A2) were performed.

### Western blot analysis

Protein lysates from muscle were resolved on 10% SDS–PAGE gels and transferred to PDVF membranes. Full size calpain‐3 and autolytic fragments were detected with the above described antibodies. Rabbit anti‐GAPDH (Sigma‐Aldrich, Ref. G9545) was developed as load control.

### *CAPN3* genetic screening

DNA was extracted from blood and screened by Sanger sequencing of the 24 exons and the flanking sequence of each intron of *CAPN3* (ABI3730 DNA Sequencer).

### Next generation sequencing

Targeted next generation sequencing of 106 neuromuscular disease genes, including 15 LGMD genes, 12 distal myopathy genes and 52 CMT genes was performed (Illumina) on both patients. To cover other genes of interest, exome sequencing was performed on patient 1 with a median coverage of 100x (Illumina) and genes included in GeneTable 2018 (http://www.musclegenetable.fr) were screened for pathogenic variants.

### Transcript analysis

RNA was extracted from frozen muscle (RNA purification kit Norgen, Ref.17200). Integrity of RNA was checked using the Agilent 2100 BioAnalyzer system. PrimeScript™ RT Master Mix kit (Takara, Ref. RR036A) was used to obtain cDNA. *CAPN3* cDNA was amplified by PCR. *DES* and *DAG1* cDNA was also amplified as controls. *CAPN3* cDNA PCR products were sequenced by Sanger method.

### Haplotype analysis

Relatedness of the two cases was studied by haplotype analysis. The two affected individuals and parents of Patient 2 were included in the analysis. No first‐degree relatives of Patient 1 were available. Six microsatellite markers spanning 13 Mb around *CAPN3* were amplified by PCR using fluorescently labeled forward primers. PCR products were analyzed in an ABI3500 Genetic Analyzer and their sizes were estimated using GeneMapper software 4.1. Additionally, five single nucleotide polymorphisms with minor allele frequency close to 50% were analyzed by Sanger sequencing.

## Results

### Clinical findings

**Figure 1:**
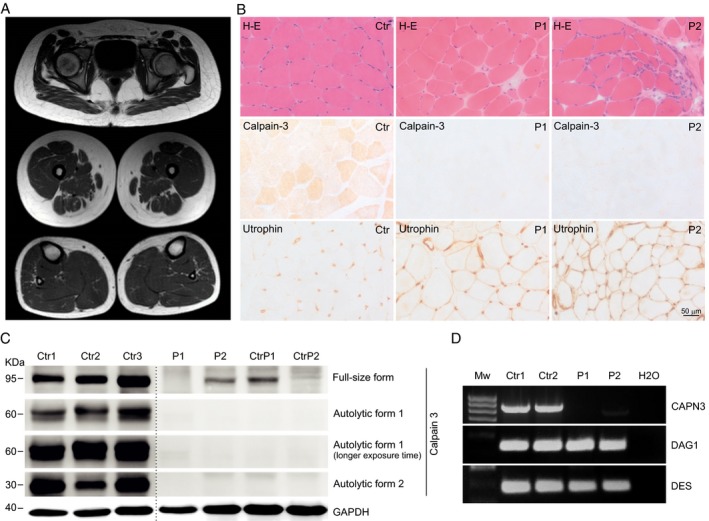
Muscle MRI, muscle biopsy, CAPN3 immunoblot and RT‐PCR in patients homozygous for *CAPN3* c.1992 + 2T>G. (A) Muscle MRI of Patient 1 showed mild fat infiltration of posterior compartment of thigh. (B) Muscle biopsy: H&E and immunohistochemical staining for calpain‐3 (2C4 antibody) and utrophin in a control, Patient 1 and Patient 2. Mild dystrophic pattern and a complete absence of immunoreactivity for calpain‐3 were present in both cases. Utrophin immunostaining showed a moderate overexpression compared with control muscle. (C) Immunoblot in muscle tissue of Patient 1 (P1) shows complete absence of the full‐size form as well as autocatalitic forms of Calpain‐3. In Patient 2 (P2), full‐size form is reduced and in both autocatalytic forms are absent, compared with Controls (Ctr). CtrP1 and CtrP2: LGMD2A disease controls (D) RT‐PCR of exons 11‐21 of CAPN3 cDNA from muscle tissue: No amplification was observed in Patient 1 (P1) and a faint band of slightly reduced molecular weight was obtained in Patient 2 (P2). RT‐PCR of control cDNAs were successfully amplified in all samples. RNA integrity numbers (RIN) obtained for the RNA samples were 8.3 (Crt1), 7.3 (Ctr2), 8.7 (P1), and 6.8 (P2).

Patient 1 was born to consanguineous parents. She was first investigated at the age of 13 after an incidental finding of high levels of serum CK (8900 IU/L). When asked, she reported low performance on sports and motor activities for the last year. Examination at age 13 showed mild proximal lower limb weakness and hypoactive deep tendon reflexes. She later developed difficulty to climb stairs and to run. Last examination at age 15 showed asymmetric scapular winging, weakness of wrist and finger extensors, and interossei and proximal lower limb weakness. A muscle MRI performed at age 13 showed mild fat infiltration of posterior compartment of thighs (Fig. 1A). Patient 2 was born to consanguineous parents. He presented at age 8 years after finding a serum CK of 3700 IU/L. He reported muscle cramps when playing sports as the only muscle symptom. At age 10 he was found to have mild proximal upper and lower limb weakness. Last examination at age 12 revealed bilateral scapular winging and waddling gate. He could stand from a seat but needed support to stand from a squat. There was weakness of deltoids, biceps, glutei, tibialis anterior, and adductor hallucis longus, being able to walk on his toes but not on his heels.

### Muscle histopathology

Muscle biopsies were performed at age 13 in Patient 1 and at age 10 in Patient 2. Both showed a mild dystrophic pattern, absence/deficiency of calpain‐3, and overexpression of utrophin (Fig. 1B).

### Immunoblot

Immunoblot performed on muscle tissue showed absence of the full‐length form of calpain‐3 in muscle from Patient 1, and severe reduction in Patient 2. Absence of autocatalytic forms was found in both patients (Fig. 1C).

### Genetic screening

**Figure 2:**
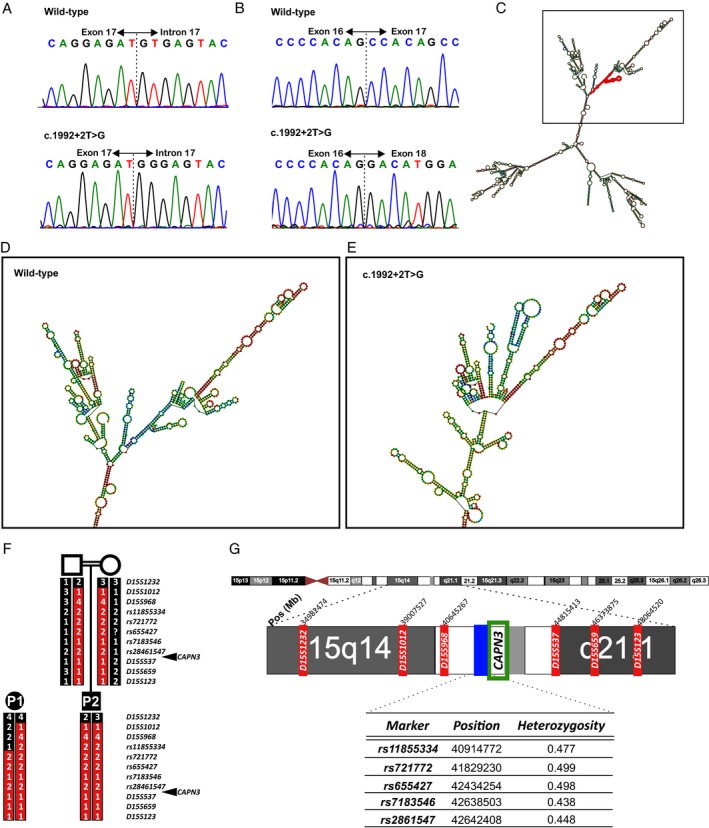
Molecular characterization of the *CAPN3* c.1992 + 2T>G variant. (A) Sanger sequencing of genomic DNA of Patient 1 showing a homozygous nucleotide substitution T to G at position + 2 of intron 17 (B) Sanger sequencing of exons 11‐21 of cDNA from muscle showing skipping of exon 17. (C, D, E) Structural changes induced in the *CAPN3* c.1992 + 2T> G mRNA molecule. (C) Predicted full‐length wild‐type *CAPN3* mRNA structure. Exon 17 is marked in red. The black box highlights the region of the mRNA that harbors the structural changes. (D, E) Magnification of the region showing of the structural change of the mRNA molecule. (D) *Wild-type CAPN3* mRNA and (E) *c.1992 + 2T>G CAPN3* mRNA . (F) Haplotype analysis showing a shared area of homozygosity and common haplotype around the position of *CAPN3*. Microsatellite markers and single nucleotides polymorphisms used for the analysis are shown. (G) Diagram of chromosome 15 showing the region studied including the position of the markers used. Position of *CAPN3* is marked in a green box. Microsatellite markers positions are highlighted in red and single nucleotide polymorphisms in blue.

Sanger sequencing of *CAPN3* identified an intronic homozygous NM_000070.2 c.1992 + 2T>G variant in both patients (Fig. 2A). Accordingly, parents of Patient 2 were heterozygous carriers. Targeted NGS performed in Patients 1 and 2 and exome sequencing in Patient 1 confirmed the presence of a homozygous *CAPN3* c.1992 + 2T>G variant and did not identify pathogenic variants in genes related with other neuromuscular disease. The *CAPN3* c.1992 + 2T>G variant is not present in databases of normal controls (gnomAD).^11^ Screening of whole exome sequencing data from 50 Roma controls did not identify any carriers. The variant involves the second position of the canonical donor splice site (GT) of intron 17, is predicted to be damaging by DANN,^12^ MutationTaster^13^ and FATHMM‐MKL^14^ and to break the donor splice site by SSPP analysis NetGene2.^15^ A pathogenic T> A variant at the same c.1992 + 2 position has been reported, although no characterization is available (https://www.ncbi.nlm.nih.gov/clinvar/variation/596001/).

### RNA analysis

To investigate whether the c.1992 + 2T>G variant affects splicing, total muscle RNA was retrotranscribed to cDNA and amplified. No *CAPN3* cDNA was detected in Patient 1. In Patient 2, severely reduced levels were observed. Sanger sequencing of cDNA in Patient 2 revealed skipping of exon 17 (Fig. 2B), predicted to cause a 25 amino‐acid in‐frame deletion. PCR amplification of control cDNAs expressed in muscle (*DES* and *DAG1*) was efficient in patients and controls (Fig. 1D).

RNAfold program (Vienna RNA package)^16^ analysis of the mutant RNA molecule predicted the introduction of strong secondary structure changes involving a large part of the molecule (Fig. 2C–E).

### Haplotype analysis

To investigate whether the identified mutation has a common ancestral origin in the two families we analysed the genotype of 11 markers spanning 13Mb which identified an area of at least 6.2Mb of homozygosity and common haplotype, around the position of the mutation, shared by the two patients, revealing a common ancestor and a founder effect (Fig. 2F–G).

## Discussion

Here, we report a novel Roma mutation causing LGMD. This is, to our knowledge, the first *CAPN3* mutation reported in the Roma population. The mutation, identified in apparently unrelated patients, has a common ancestor supporting the notion that this is a Roma founder mutation. Analysis of subsequent cases would be necessary to corroborate this founder effect.

Our patients displayed distal weakness from early in disease course. The clinical presentation of *CAPN3* mutations is highly variable, including asymptomatic HyperCK, pelvic girdle weakness and scapulohumeral weakness.^17^ Distal muscle involvement, although rare, have been previously described.^18^, ^19^ However, to our knowledge, involvement of distal upper limb muscles at early stages has not been documented before. Other intronic mutations in *CAPN3* have previously been reported causing abnormal splicing,^20^, ^21^ including a c.1992 + 1 G> T *CAPN3* variant that lies on a base next to our novel mutation.^22^, ^23^ The c.1992 + 1 G> T *CAPN3* mutation abolishes the canonical donor splice site of exon 17, causing skipping of exon 17 or inclusion of 31bp of intron 17 resulting in very low or undetectable levels of muscle CAPN3 RNA and protein,^22^, ^23^ in line with our results.

RNA quality control systems identify and degrade aberrant mRNAs through different mRNA surveillance pathways.^24^ Non‐sense^25^, ^26^ and non‐stop^19^ mediated decay (NMD, NSD) are mechanisms to trigger RNA degradation. The most recently known RNA surveillance mechanism is No‐Go Decay (NGD), in which aberrant secondary structures causing stalls in translation elongation trigger mRNA endonucleolytic cleavage.^27^ NGD has been described as the pathogenic mechanism in other splice site mutation, causing human disease.^28^

Different levels of protein detected by immunoblot among patients with the same *CAPN3* mutation have previously been described,^29^ however, it is interesting that in our report, different protein and RNA levels are seen in patients of the same background, both being equally clinically affected from a young age and sharing a characteristic phenotype with distal involvement. The different RNA and protein levels observed may represent unequal efficiency in the RNA degradation mechanisms, which are not completely understood.

We have shown how the *CAPN3* c.1992 + 2T>G induces structural changes in the mutant RNA. These profound structural alterations of the mRNA molecule may cause stalls during translation and elongation. In our patients, RNA is severely reduced or absent. These findings are in keeping with no‐go mediated decay as a novel pathogenic mechanism in calpainopathies.

## Conflict of interest

The authors declare no conflicts of interest.

## Author Contributions

F.M., C.P., and M.C.‐S. were responsible for the conception and design of this study. M.M.‐G. and M.C.‐S. assessed the patients, provided clinical information and collected biological samples. E.R., E.S.‐M. and R.A.‐P. processed and studied muscle biopsy and western blot. F.M., I.M., and FJ.M performed the genetic analysis and haplotype. F.M. performed molecular and *in silico* analysis. F.M., C.P., and M.C.‐S. drafted the manuscript and figures. M.C.‐S. coordinated all the study. All authors read and approved the final manuscript.
